# Supplementary material for: Long-Term Outcomes of Ledipasvir/Sofosbuvir Treatment in Hepatitis C: Viral Suppression, Hepatocellular Carcinoma, and Mortality in Mongolia
Source: Viruses. 2025 May 22;17(6):743. doi: 10.3390/v17060743 (PMC12197668; doi:10.3390/v17060743)
Supplement: Supplementary file 1 [file viruses-17-00743-s001.zip › viruses-3583500-supplementary.pdf]

Table S1. Baseline characteristics of the study population by follow-up status.

Findings	HCV-RNA detection		P-value
	Retained	Lost-to FU	
**Age (years)**	52.4 (45.2–53.3)	51.9 (45.1–55.1)	0.003
Male sex	103 (34.2%)	80 (39.6%)	0.234
Liver cirrhosis, n (%)	53 (17.6%)	24 (11.8%)	0.111
ALT	30.1 (28.2–32.1)	30.3 (25.3–33.7)	<0.01
AST	30.2 (28.2–32.3)	31.0 (26.1–31.2)	<0.01
PLT	204.5 (191–218)	206.3 (201–209)	<0.01
APRI score	0.48 (0.42–0.55)	0.49 (0.44–0.61)	<0.01
FIB-4 score	1.53 (1.38–1.67)	1.52 (1.19–1.60)	<0.01

Data are presented as mean ± SD.
